# Supplementary material for: A combined opposite targeting of p110δ PI3K and RhoA abrogates skin cancer
Source: Commun Biol. 2024 Jan 5;7:26. doi: 10.1038/s42003-023-05639-8 (PMC10770346; doi:10.1038/s42003-023-05639-8)
Supplement: Supplementary file 2 — Supplementary Information [file 42003_2023_5639_MOESM2_ESM.pdf]

Supplementary Information

A combined opposite targeting of p110δ PI3K and RhoA abrogates skin cancer

Niki Tzenaki[1], Lydia Xenou[1], Evangelia Goulielmaki[1], Anna Tsapara[1], Irene Voudouri[1], Angelika Antoniou[1], George Valianatos[1], Maria Tzardi[2], Eelco De Bree[3], Aikaterini Berdiaki[4], Antonios Makrigiannakis[4] and Evangelia A. Papakonstanti[1*]

[1]Department of Biochemistry, School of Medicine, University of Crete, Heraklion, Greece

[2]Department of Pathology, School of Medicine, University of Crete, University Hospital, Heraklion, Greece

[3]Department of Surgical Oncology, School of Medicine, University of Crete, University Hospital, Heraklion, Greece

[4]Department of Obstetrics and Gynaecology, School of Medicine, University of Crete, University Hospital, Heraklion, Greece

[*]**Corresponding author:**

Evangelia Papakonstanti
Faculty of Medicine (room 2B-14)
University of Crete
Vassilika Vouton
GR-71003
Heraklion-Crete
Greece
Tel: +30-2810-394554
Email: epapak@uoc.gr

Supplementary Figure 1

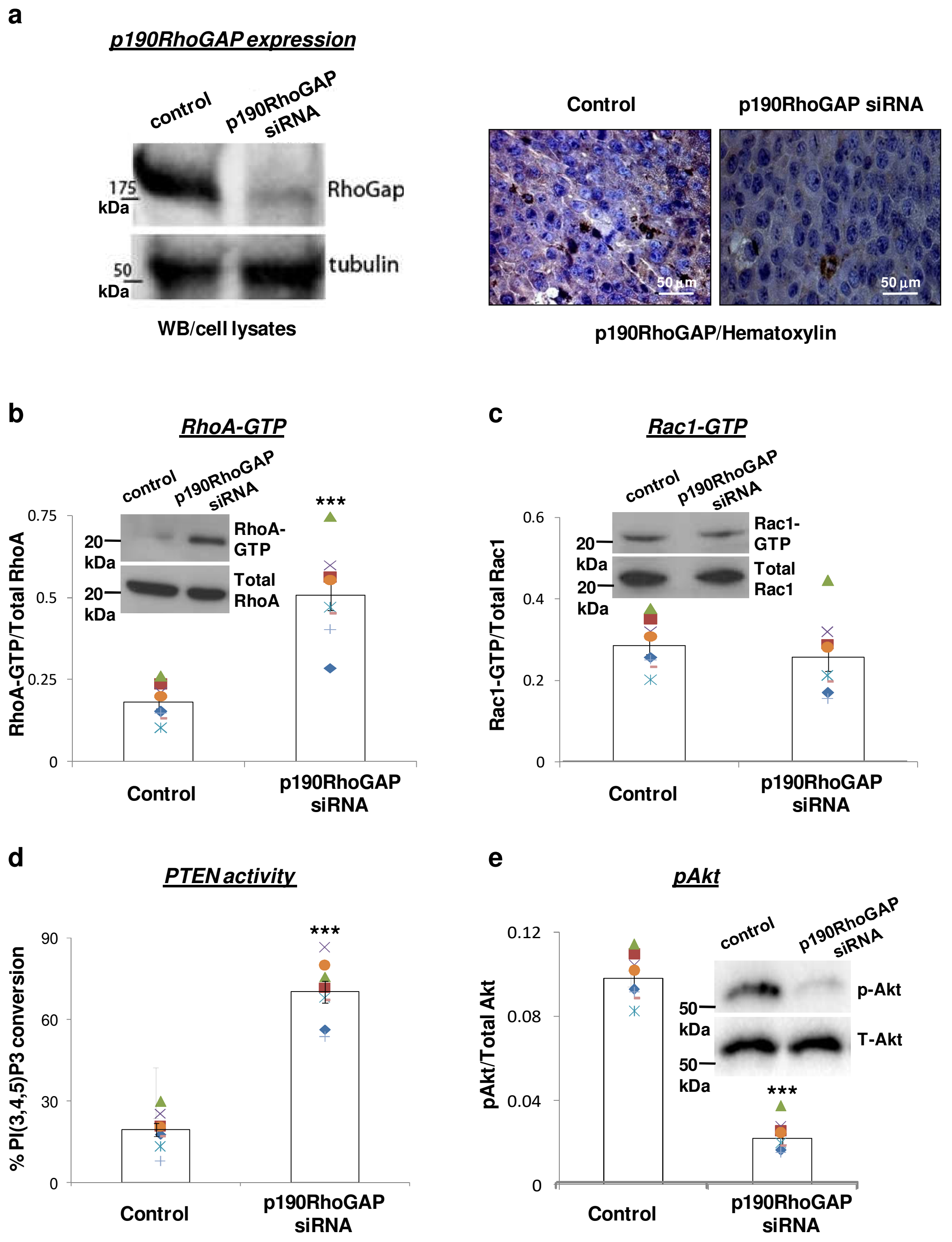

Supplementary Figure 1/ Suppression of p190RhoGAP expression by siRNA silencing induces PTEN activity and RhoA-GTP but not Rac1-GTP levels and reduces the phosphorylation of Akt into B16 melanoma tumours. Impact of intratumoural injection of p190RhoGAP siRNA into melanoma tumours on p190RhoGAP protein expression (**a**), RhoA-GTP levels (**b**), Rac1-GTP levels (**c**), activity of PTEN (**d**) and phosphorylation of Akt (**e**) in tumour cells excised from B16 tumour bearing mice (n=8 mice/group). Scale bar= 50 µm. The tubulin, total RhoA, total Rac1 and total Akt bands presented for normalization were derived from the same respective membranes. All graphs represent means±s.e.m. Each symbol on the different groups denotes data from a different animal of the respective group. Statistically significant differences are indicated by *** (P < 0.001), as determined by the Mann-Whitney U test.

Supplementary Figure 2

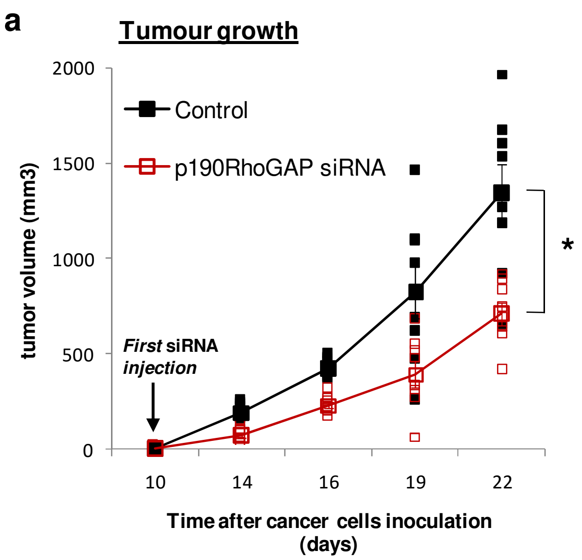

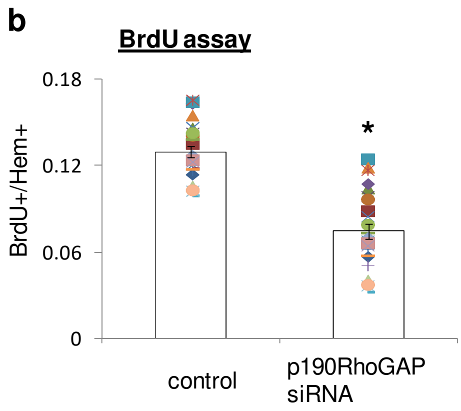

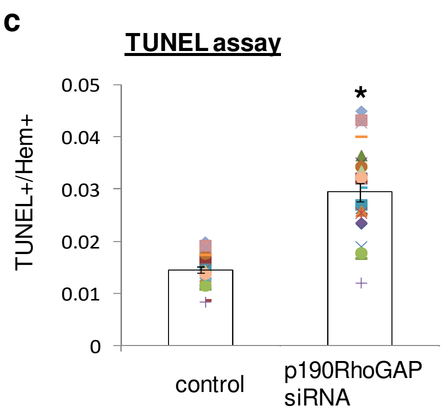

Supplementary Figure 2/ Intratumoural administration of p190RhoGAP siRNA reduces tumour growth. a, NSG mice were inoculated with B16 cells on day 0 and treated with intratumoural injections of p190RhoGAP siRNA or control on day +10 and on every other day until the end of the experiment. Tumour growth was measured. n=8 mice/group. Each symbol on the different groups denotes data from a different animal of the respective group. **b,** Comparison of BrdU-positive cells in tumours from mice that were treated with intratumoural injections of p190RhoGAP siRNA and mice treated with intravenous injections of control. Each symbol on the different groups denotes data from 8 fields/ section of 3 sections of stained cells. **c,** Comparison of TUNEL-positive cells in tumours from mice that were treated with intratumoural injections of p190RhoGAP siRNA and mice treated with intravenous injections of control. Each symbol on the different groups denotes data from 8 fields/ section of 3 sections of stained cells. All graphs represent means±s.e.m. Statistically significant differences are indicated by * ($P < 0.1$) as determined by the Mann-Whitney U test.

Supplementary Figure 3

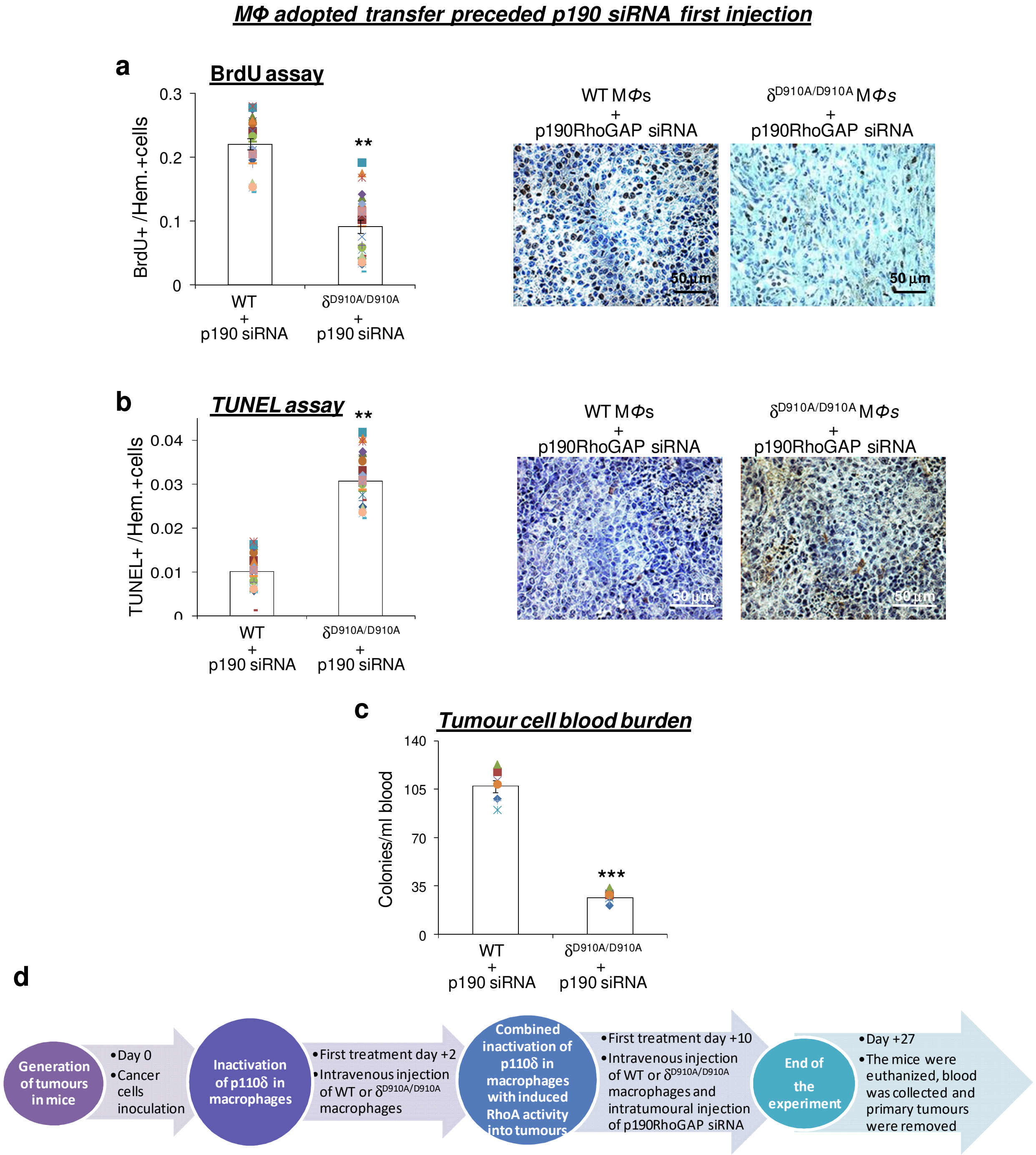

Supplementary Figure 3/ Impact of the opposite targeting of p110δ and RhoA on the proliferation, apoptosis and metastasis of melanoma tumour cells when the intravenous injections of macrophages preceded the intratumoural injections of p190RhoGAP siRNA. a, Cell proliferation in tumours excised from mice that were first treated (starting on day +2) with intravenous injections of WT or $\delta^{D910A/D910A}$ macrophages and then with intratumoural injections of p190RhoGAP siRNA (starting on day +10) was determined by BrdU incorporation (brown spots) (right panels). Scale bar=50 µm. Comparison of BrdU-positive cells in tumours from mice that were treated with intravenous injections of WT macrophages and intratumoural injections of p190RhoGAP siRNA and mice treated with intravenous injections of $\delta^{D910A/D910A}$ macrophages and intratumoural injections of p190RhoGAP siRNA (left panel). Each symbol on the different groups denotes data from 8 fields/ section of 3 sections of stained cells. **b,** Apoptosis in tumours excised from mice that were first treated (starting on day +2) with intravenous injections of WT or $\delta^{D910A/D910A}$ macrophages and then with intratumoural injections of p190RhoGAP siRNA (starting on day +10) was determined by TUNEL assay (brown spots) (right panels). Scale bar=50 µm. Comparison of TUNEL positive cells in tumours from mice that were treated with intravenous injections of WT macrophages and intratumoural injections of p190RhoGAP siRNA and mice treated with intravenous injections of $\delta^{D910A/D910A}$ macrophages and intratumoural injections of p190RhoGAP siRNA (left panel). Each symbol on the different groups denotes data from 8 fields/ section of 3 sections of stained cells. **c,** Intravasation efficiency of cancer cells as determined by tumour cells blood burden at the end point of the experiments in NSG mice which received WT or $\delta^{D910A/D910A}$ macrophages (starting on day +2) and then intratumoural injections of p190RhoGAP siRNA (starting on day +10). All immunostainings were performed on tumour sections from tumours excised at the end point of each experiment. Each symbol on the different groups denotes data from a different animal of the respective group. All graphs represent means±s.e.m. Statistically significant differences are indicated by ** ($P < 0.01$) or *** ($P < 0.001$), as determined by the Mann-Whitney U test. **d**, Graphical representation of experimental events chronologically.

Supplementary Figure 4

MΦ adopted transfer *followed* p190 siRNA first injection

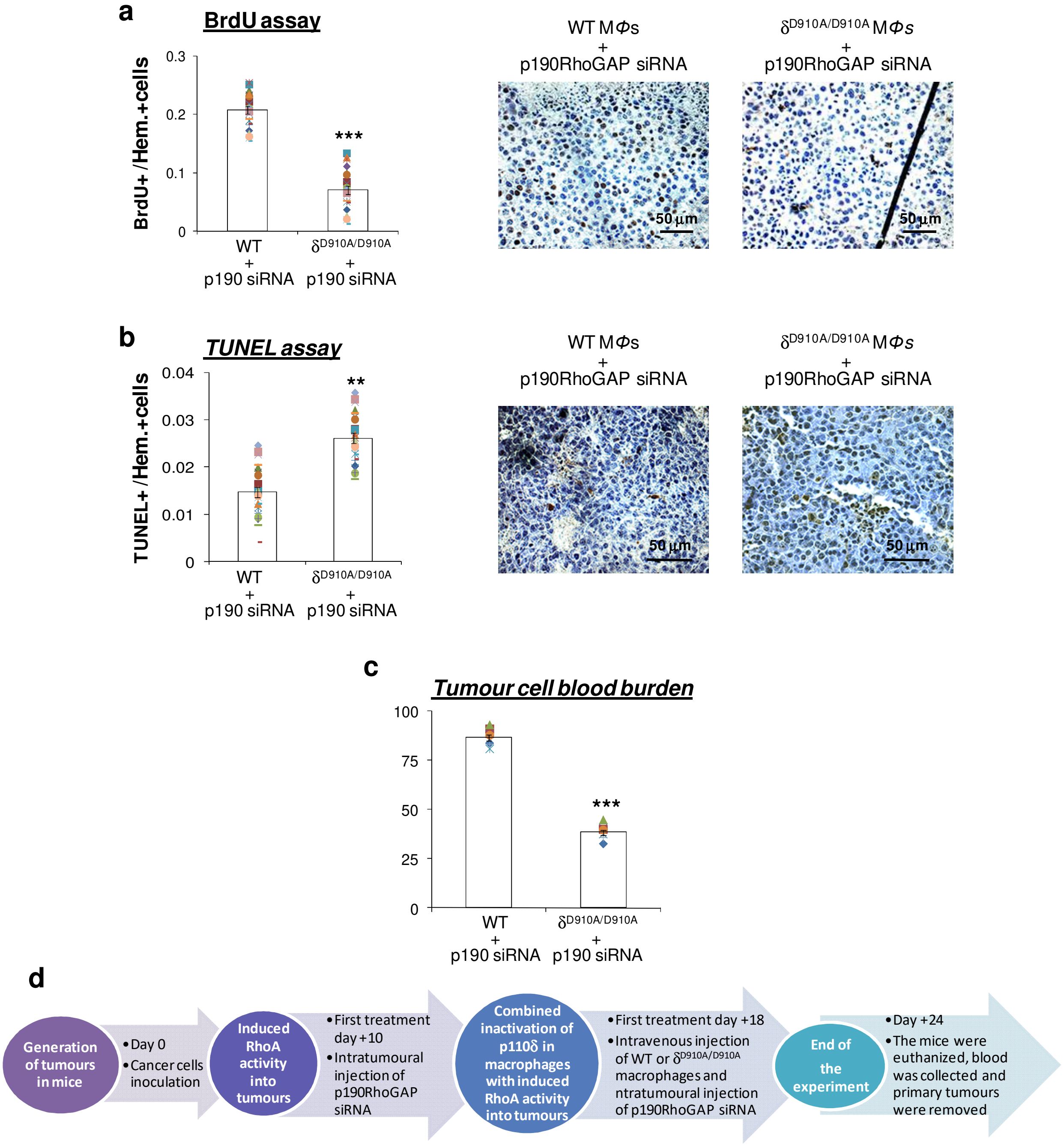

Supplementary Figure 4/ Impact of the opposite targeting of p110δ and RhoA on the proliferation, apoptosis and metastasis of melanoma tumour cells when the intravenous injections of macrophages followed the intratumoural injections of p190RhoGAP siRNA. a, Cell proliferation in tumours excised from mice that were first treated (starting on day +10) with intratumoural injections of p190RhoGAP siRNA and then with intravenous injections of WT or $\delta^{D910A/D910A}$ macrophages (starting on day +18) was determined by BrdU incorporation (brown spots) (right panels). Scale bar=50 µm. Comparison of BrdU-positive cells in tumours from mice that were treated with intravenous injections of WT macrophages and intratumoural injections of p190RhoGAP siRNA and mice treated with intravenous injections of $\delta^{D910A/D910A}$ macrophages and intratumoural injections of p190RhoGAP siRNA (left panel). Each symbol on the different groups denotes data from 8 fields/ section of 3 sections of stained cells. **b,** Apoptosis in tumours excised from mice that were first treated (starting on day +10) with intratumoural injections of p190RhoGAP siRNA and then with intravenous injections of WT or $\delta^{D910A/D910A}$ macrophages (starting on day +18) was determined by TUNEL assay (brown spots) (right panels). Scale bar=50 µm. Comparison of TUNEL positive cells in tumours from mice that were treated with intravenous injections of WT macrophages and intratumoural injections of p190RhoGAP siRNA and mice treated with intravenous injections of $\delta^{D910A/D910A}$ macrophages and intratumoural injections of p190RhoGAP siRNA (left panel). Each symbol on the different groups denotes data from 8 fields/ section of 3 sections of stained cells. **c,** Intravasation efficiency of cancer cells as determined by tumour cells blood burden at the end point of the experiments in NSG mice which received intratumoural injections of p190RhoGAP siRNA (starting on day +10) and then WT or $\delta^{D910A/D910A}$ macrophages (starting on day +18). All immunostainings were performed on tumour sections from tumours excised at the end point of each experiment. Each symbol on the different groups denotes data from a different animal of the respective group. All graphs represent means±s.e.m. Statistically significant differences are indicated by ** ($P < 0.01$), as determined by the Mann-Whitney U test. **d**, Graphical representation of experimental events chronologically.

Supplementary Figure 5

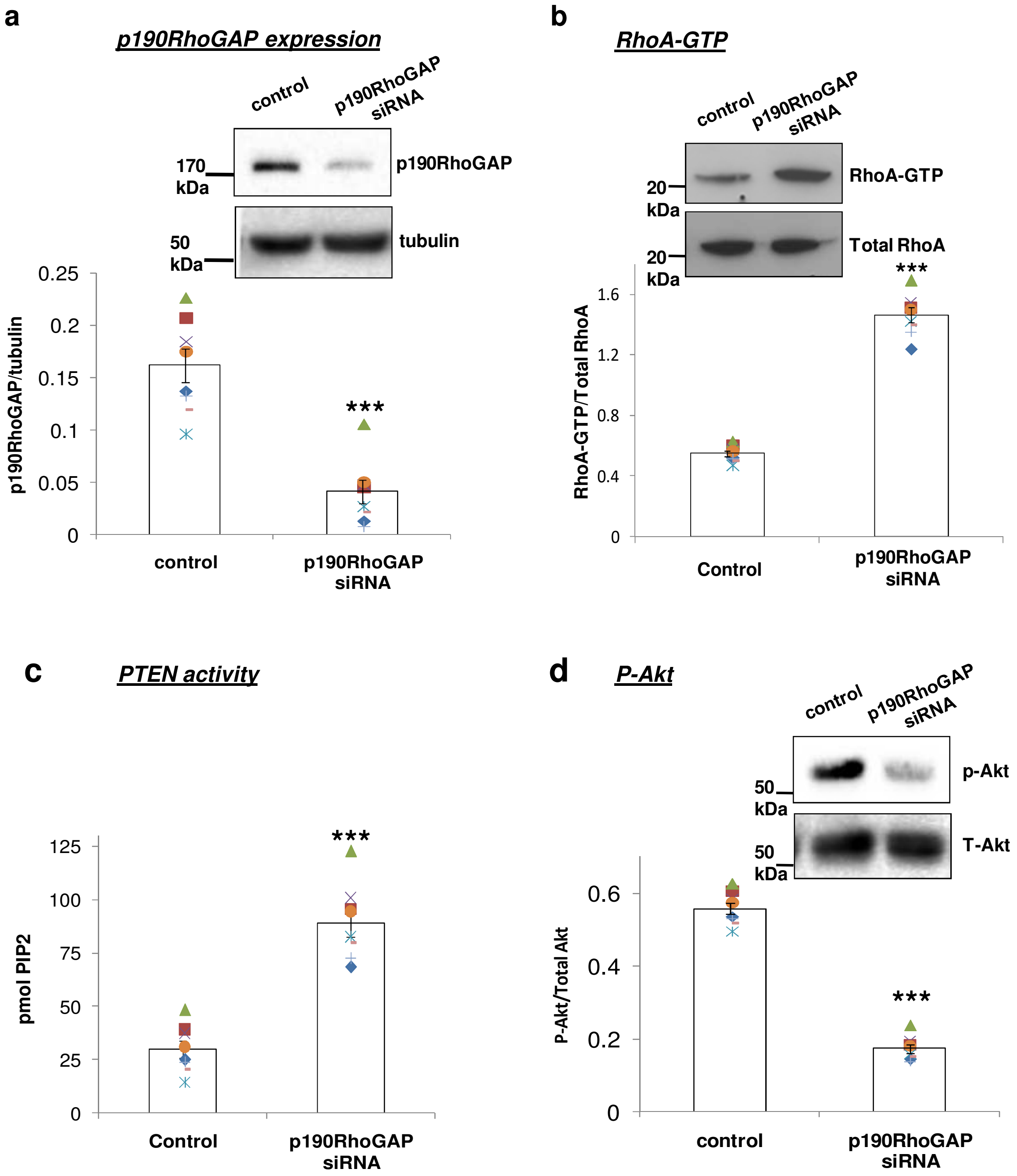

Supplementary Figure 5/ Suppression of p190RhoGAP expression by siRNA silencing induces the RhoA-GTP levels and PTEN activity and reduces the phosphorylation of Akt into 451Lu human melanoma tumours. Impact of intratumoural injection of p190RhoGAP siRNA into melanoma tumours on p190RhoGAP protein expression (**a**), activity of RhoA (**b**) and PTEN (**c**) and phosphorylation of Akt (**d**) in tumour cells excised from 451Lu tumour bearing mice. (n=8 mice/group). The bands of tubulin and total RhoA presented for normalization in **a** and **b** were derived from different membranes but from the same cell lysates whereas in **d** the bands of total Akt were derived from the same membrane. All graphs represent means±s.e.m. Each symbol on the different groups denotes data from a different animal of the respective group. Statistically significant differences are indicated by *** (P < 0.001), as determined by the Mann-Whitney U test.

Supplementary Figure 6

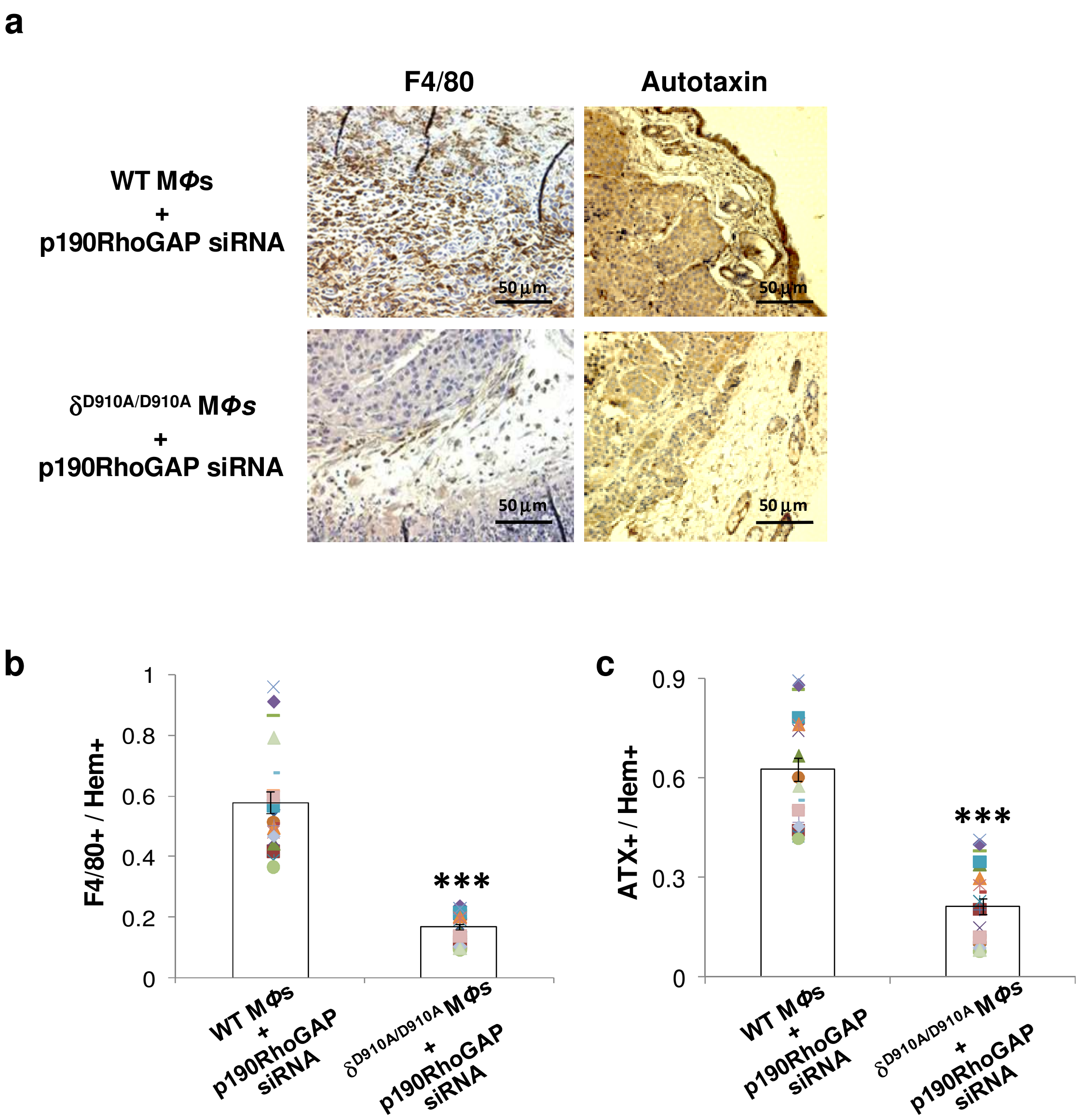

Supplementary Figure 6/ Impact of the opposite targeting of p110δ and RhoA on the recruitment of macrophages to tumour sites and on ATX expression in 451Lu human melanoma tumours. a, Representative images of immunohistochemical staining with anti-F4/80 antibody (left panels) or anti-ATX antibody (right panels) and Hematoxylin (blue) in representative sections of 451Lu tumours from NSG mice received intravenous injections of WT macrophages and intratumoural injections of p190RhoGAP siRNA or intravenous injections of $\delta^{D910A/D910A}$ macrophages and intratumoural injections of p190RhoGAP siRNA. n=8 mice/group. Scale bar=50 µm. **b,** Comparison of F4/80-positive cells in tumours from mice received intravenous injections of WT macrophages and intratumoural injections of p190RhoGAP siRNA or intravenous injections of $\delta^{D910A/D910A}$ macrophages and intratumoural injections of p190RhoGAP siRNA. **c,** Comparison of ATX-positive cells in tumours from mice received intravenous injections of WT macrophages and intratumoural injections of p190RhoGAP siRNA or intravenous injections of $\delta^{D910A/D910A}$ macrophages and intratumoural injections of p190RhoGAP siRNA. All immunostainings were performed on tumour sections from tumours excised at the end point of each experiment. All graphs represent the mean±s.e.m. of three separate experiments. Each symbol on the different groups denotes data from 7 fields/ section of 3 sections of stained cells. Statistically significant differences are indicated by *** ($P < 0.001$), as determined by the Mann-Whitney U test.

Supplementary Figure 7

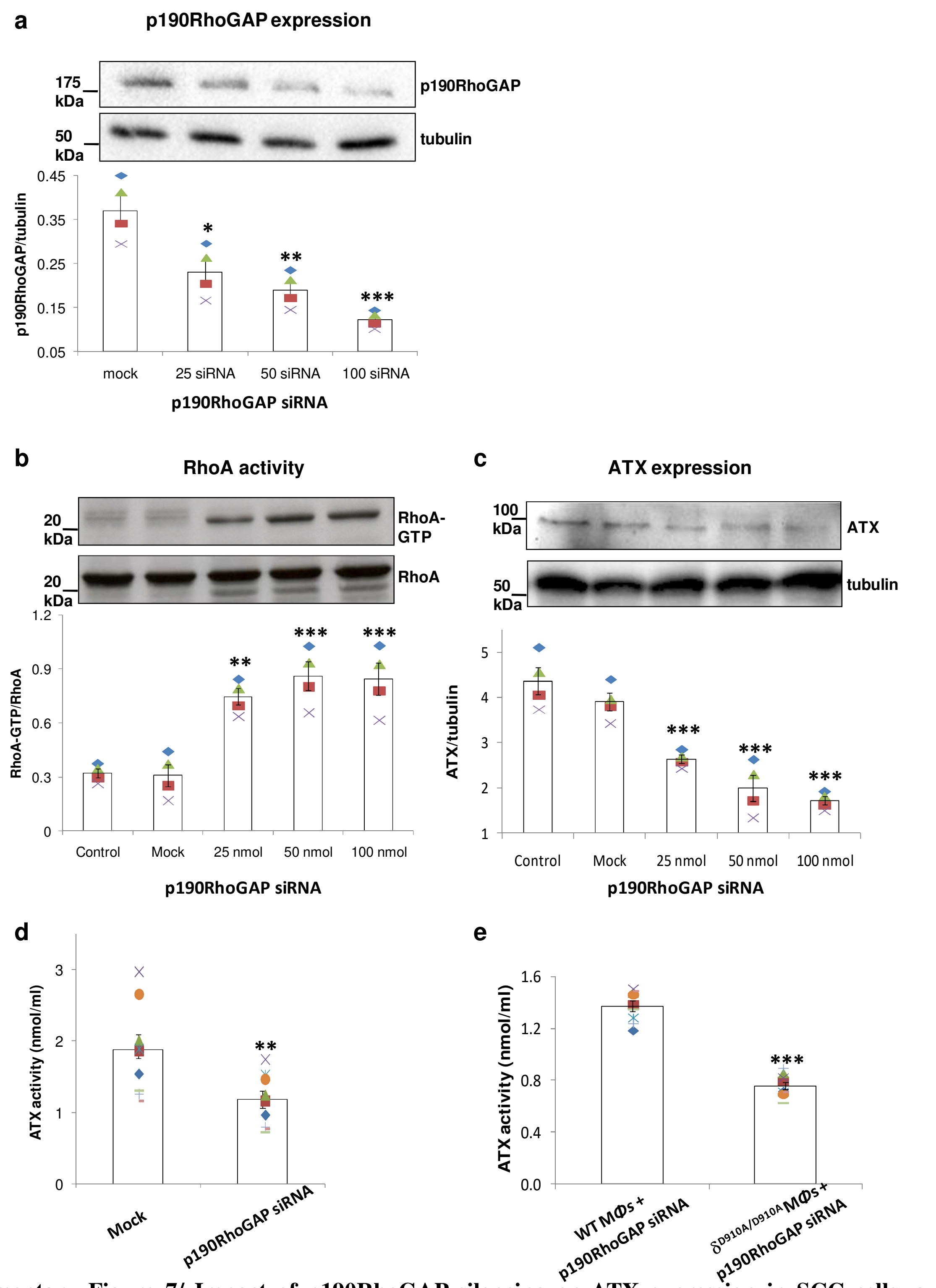

Supplementary Figure 7/ Impact of p190RhoGAP silencing on ATX expression in SCC cells. a, A431 SCC cells were transfected with different amounts of p190RhoGAP siRNA and the expression of p190RhoGAP was determined. **b,** p190RhoGAP siRNA dose dependent increase of RhoA-GTP in SCC cells. **c,** p190RhoGAP siRNA dose dependent decrease of ATX expression in SCC cells. **d,** A431 SCC cells were transfected with 50 nmol of p190RhoGAP siRNA and the activity of ATX in the culture medium was determined. **e,** ATX activity in the plasma from mice which received WT or $\delta^{D910A/D910A}$ macrophages and intratumoural injections of p190RhoGAP siRNA. The tubulin and total RhoA bands presented for normalization were derived from the same respective membranes. All graphs represent the mean±s.e.m. of at least three separate experiments. Each symbol on the different groups denotes data from at least three different experiments. Statistically significant differences are indicated by * (P < 0.5) or ** (P < 0.01) or *** (P < 0.001), as determined by the Mann-Whitney U test. In panels A to D, p190RhoGAP siRNA transfected cells were compared to mock transfected cells.

Supplementary Figure 8

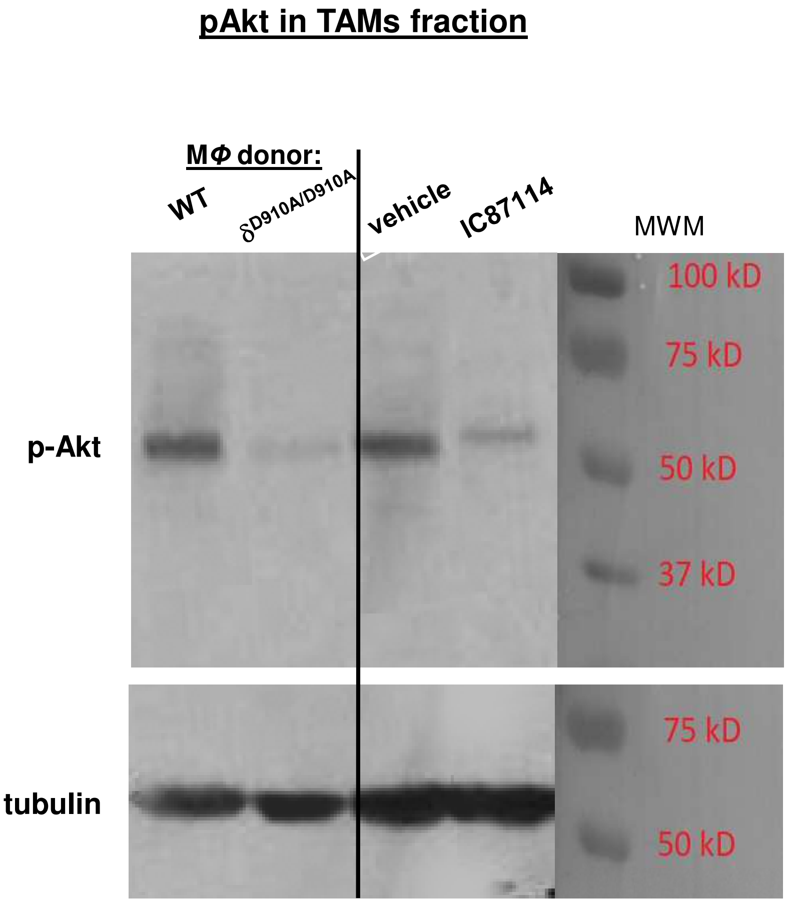

Supplementary Figure 8/ Unedited blots for Figure 1e (left) and 1f(right)

Supplementary Figure 9

a

Inhibition of ATX activity

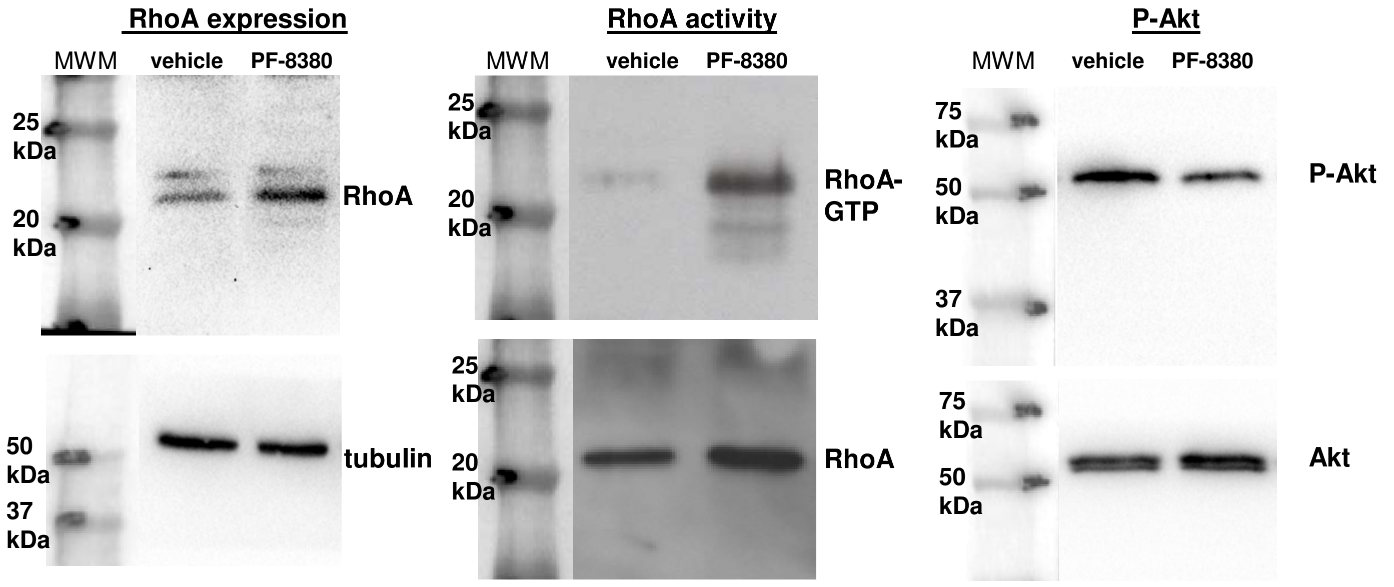

b

Inhibition of ATX activity

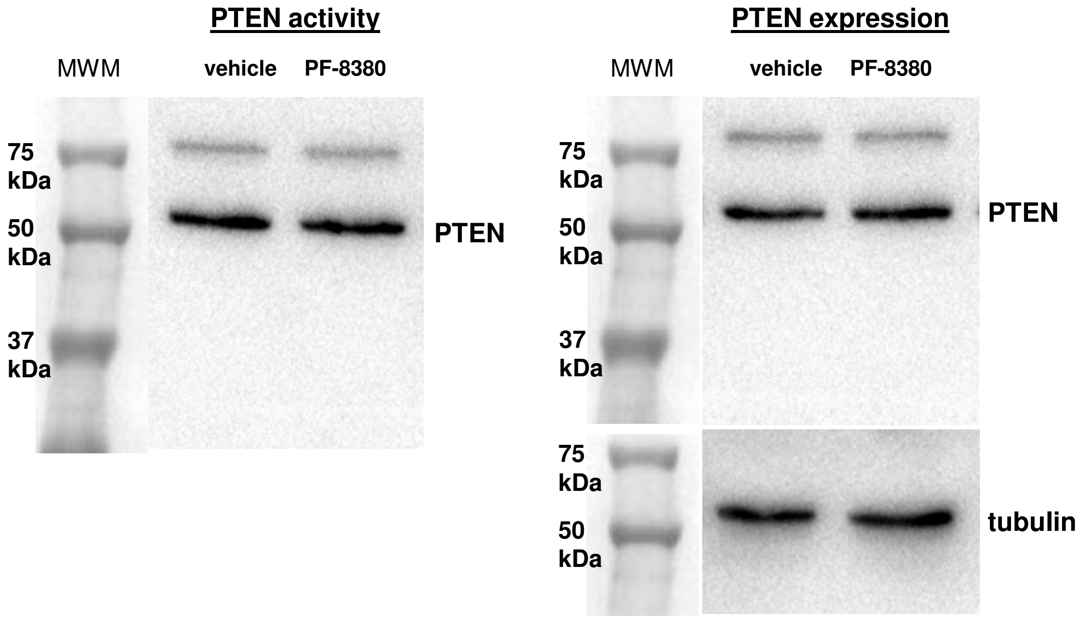

Supplementary Figure 9/ Unedited blots for Figure 7a-b

Supplementary Figure 10

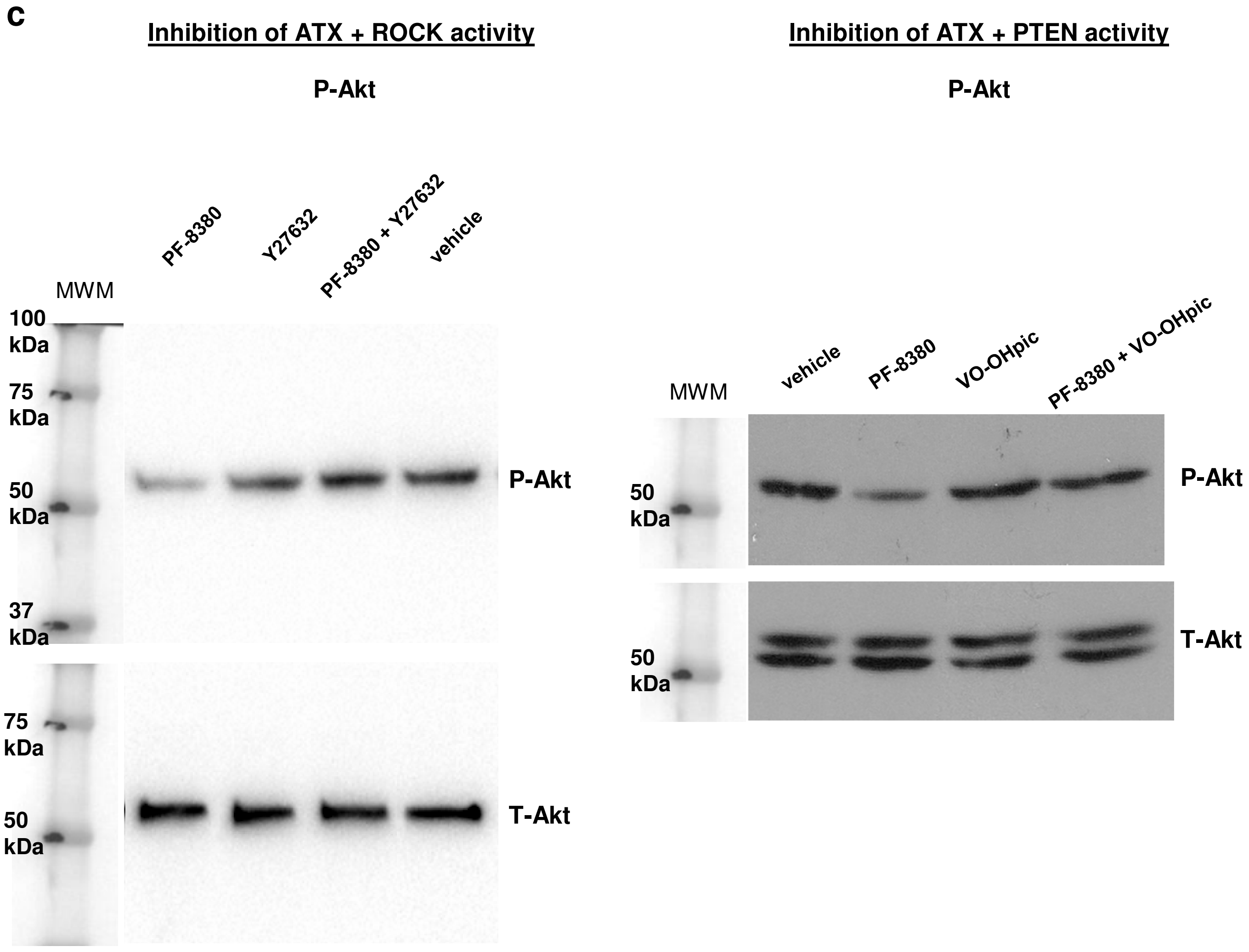

Supplementary Figure 10/ Unedited blots for Figure 7c

Supplementary Figure 11

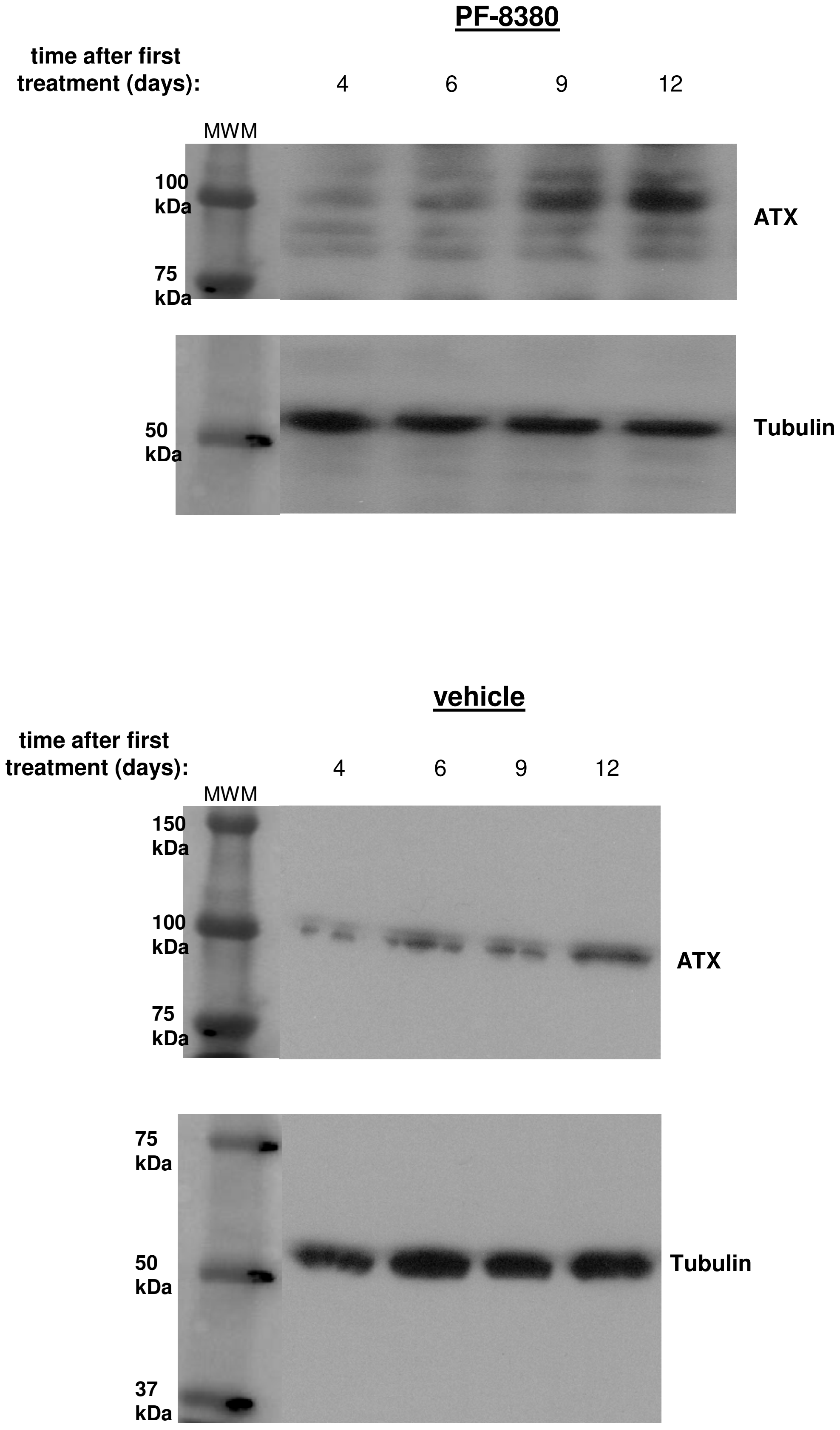

Supplementary Figure 11/ Unedited blots for Figure 8b

Supplementary Figure 12

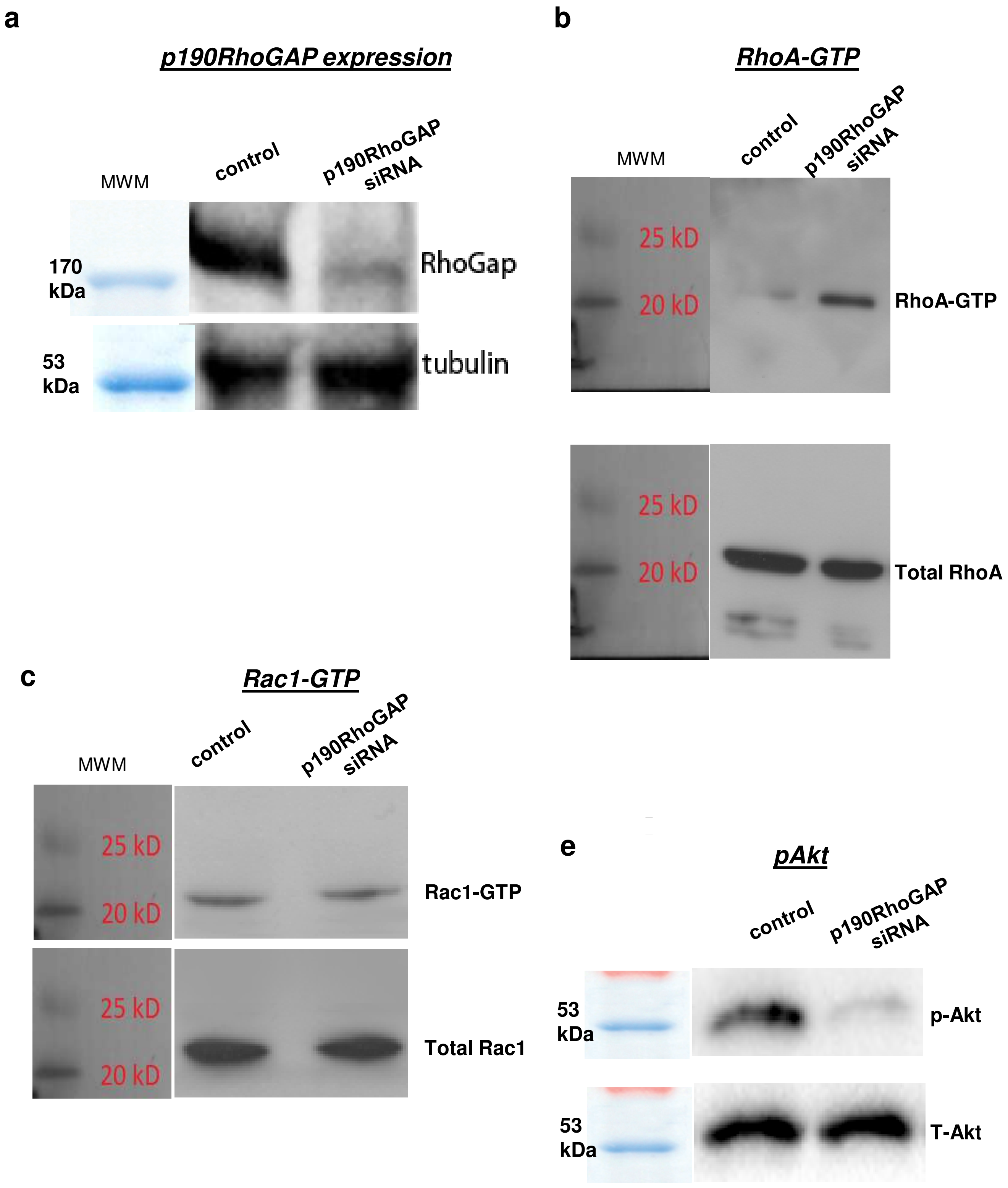

Supplementary Figure 12/ Unedited blots for Supplementary Figure 1b-c and 1e. The scans of uncropped blots for Supplementary Figure 1a were not available.

Supplementary Figure 13

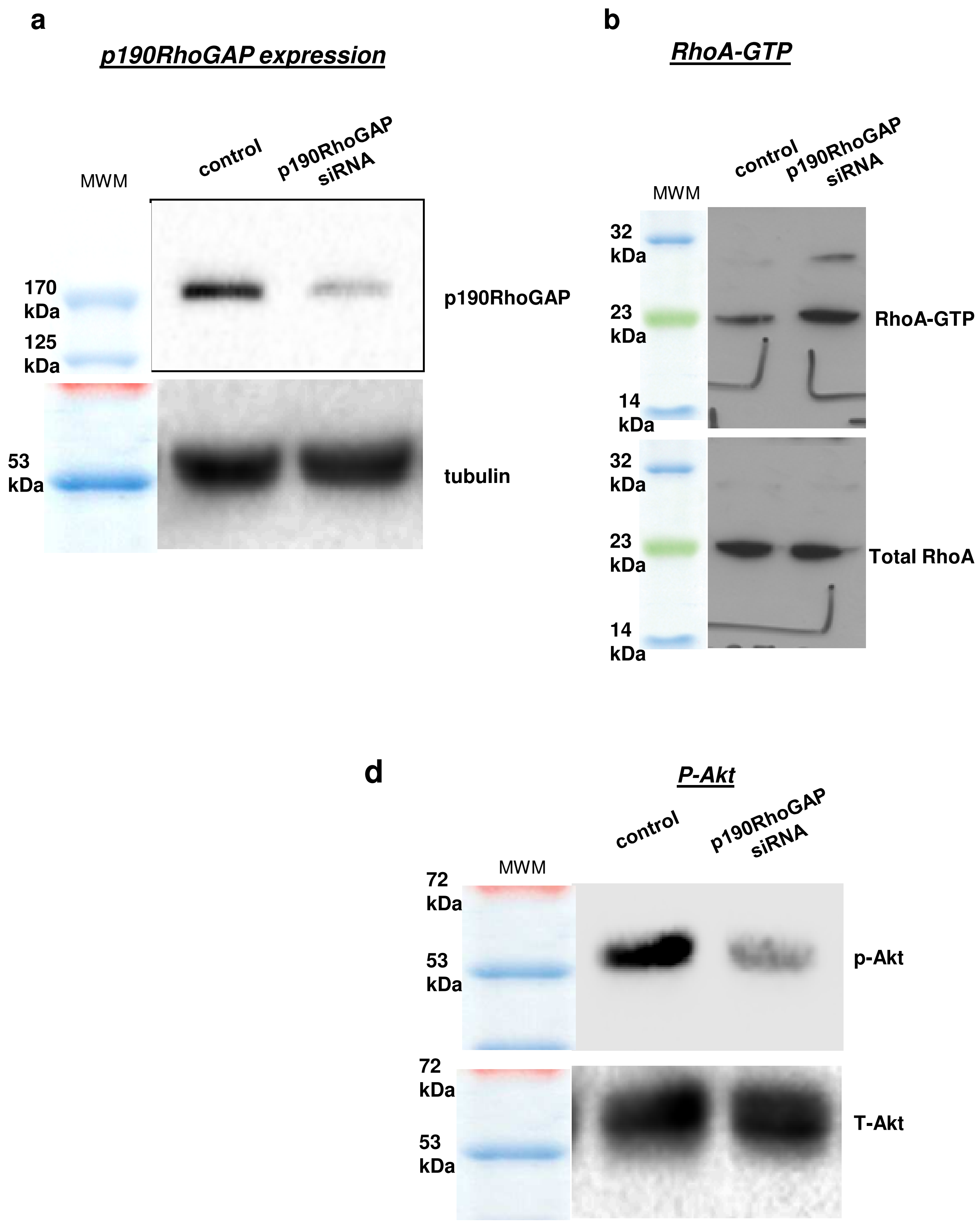

Supplementary Figure 13/ Unedited blots for Supplementary Figure 5a-b and 5d

Supplementary Figure 14

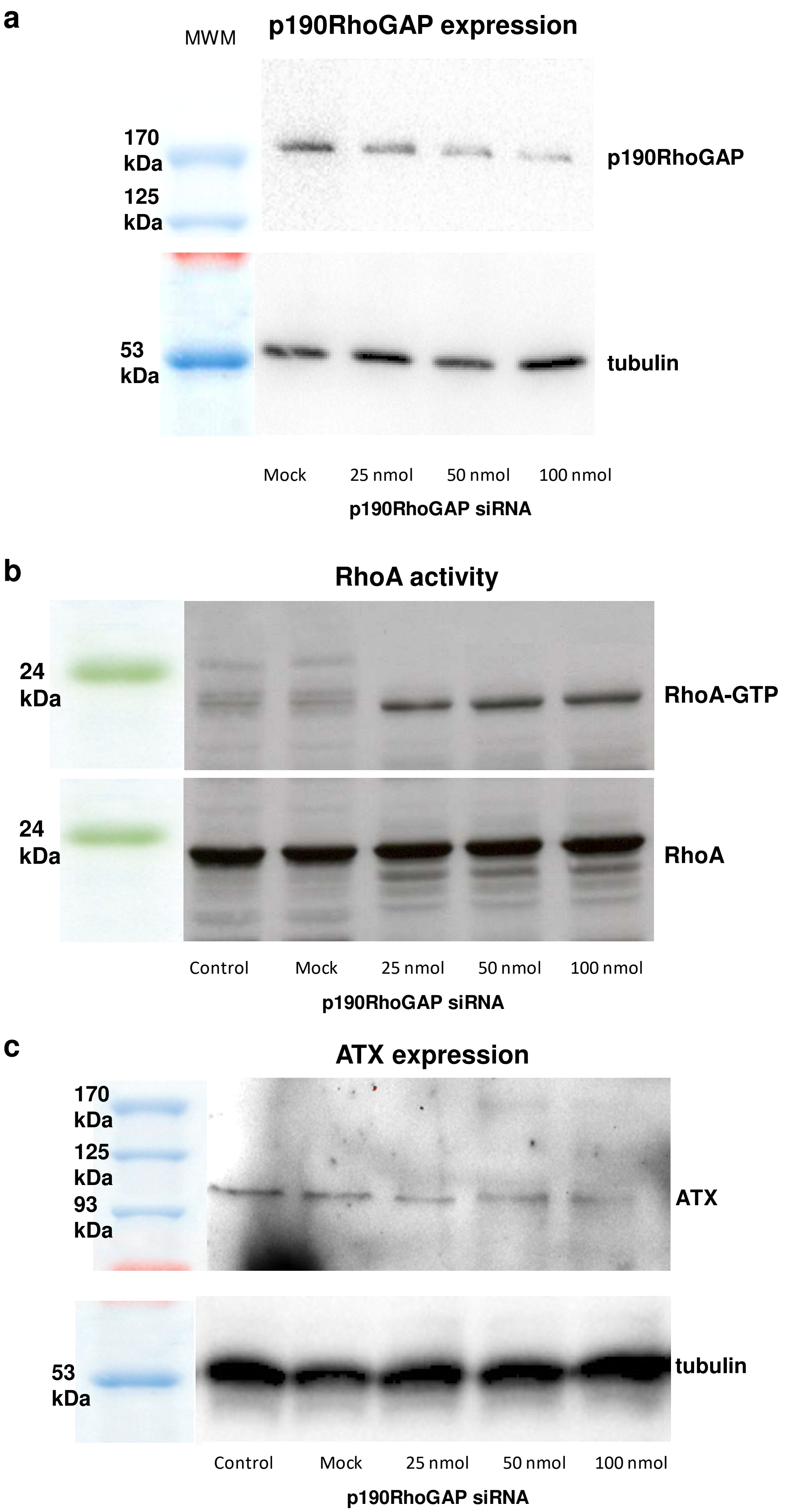

Supplementary Figure 14/ Unedited blots for Supplementary Figure 7a-c
