# Supplementary material for: A combined opposite targeting of p110δ PI3K and RhoA abrogates skin cancer
Source: Commun Biol. 2024 Jan 5;7:26. doi: 10.1038/s42003-023-05639-8 (PMC10770346; doi:10.1038/s42003-023-05639-8)
Supplement: Supplementary file 3 — Description of Additional Supplementary Files [file 42003_2023_5639_MOESM3_ESM.docx]

**Description of Additional Supplementary Files**

**File name:** Supplementary Data 1

**Description:** The source data behind the graphs in the paper; The sheets of the excel file correspond to figures’ numbers and each sheet contains the data of the respective figure.
